# Irinotecan (CPT-11) Treatment Induces Mild Gonadotoxicity

**DOI:** 10.3389/frph.2022.812053

**Published:** 2022-03-14

**Authors:** Mattan Levi, Irit Ben-Aharon, Ruth Shalgi

**Affiliations:** ^1^Department of Cell and Developmental Biology, Sackler Faculty of Medicine, Tel Aviv University, Tel Aviv, Israel; ^2^IVF Unit, Meir Medical Center, Kfar Saba, Israel; ^3^Division of Oncology, Rambam Health Care Campus, Haifa, Israel; ^4^Rappaport Faculty of Medicine, Technion, Haifa, Israel

**Keywords:** fertility, irinotecan, CPT-11, gonadotoxicity, testis, ovary

## Abstract

**Background:**

Gonadal toxicity following chemotherapy is an important issue among the population of young cancer survivors. The inhibitor of DNA topoisomerase I, irinotecan (CPT-11), is widely used for several cancer types. However, little is known about the effect of irinotecan on the fertility of both genders. Thus, the aim of the present study was to evaluate irinotecan gonadotoxicity, using a mouse model.

**Methods:**

Mature male and female mice were injected intraperitoneally with either saline (), irinotecan (100 mg/kg) or cyclophosphamide (100 mg/kg); and sacrificed one week or three months later for an acute or long-term toxicity assessment, respectively. We used thorough and advanced fertility assessment by already established methods: Gonadal and epididymal weights, as well as sperm count and sperm motility were determined; serum anti-Müllerian hormone (AMH) was measured by ELISA. Immunohistochemistry (Ki-67), immunofluorescence (PCNA, CD34), terminal transferase-mediated deoxyuridine 5-triphosphate nick-end labeling (TUNEL) and computerized analysis were performed to examine gonadal proliferation, apoptosis and vascularization. qPCR was used to assess the amount of testicular spermatogonia (Id4 and Gafra1 mRNA) and ovarian primordial oocytes reserves (Sohlh2, Nobox and Figla mRNA).

**Results:**

Females: Irinotecan administration induced acute ovarian apoptosis and decreased vascularity, as well as a mild, statistically significant, long-term decrease in the number of growing follicles, ovarian weight, and ovarian reserve. Males: Irinotecan administration caused an acute testicular apoptosis and reduced testicular spermatogenesis, but had no effect on vascularity. Irinotecan induced long-term decrease of testicular weight, sperm count and testicular spermatogonia and caused elevated serum AMH.

**Conclusion:**

Our findings imply a mild, though irreversible effect of irinotecan on mice gonads.

## Introduction

Irinotecan (7-Ethyl-10-[4-(1-piperidino)-1-piperidino] carbonyloxycamptothecin; CPT-11), alone or in combination with other drugs, has been used clinically, showing high response rates in the setting of uterine cancer, ovarian cancer, lung cancer, gastric cancer and malignant lymphoma (1). Irinotecan is a pro-drug, which is bio-activated to 7-ethyl-10-hydroxy-camptothecin (SN-38) by carboxylesterase, located mainly in the serum of rats and mice and in the liver of humans (2). SN-38 is detoxified to SN-38 glucuronide by the polymorphic enzyme uridine diphosphate glucuronosyltransferase isoform 1A1 (2). Irinotecan and its more active derivative, SN-38, is a powerful S phase-specific inhibitor of DNA topoisomerase I, a key nuclear enzyme for the relaxation of DNA double helix super-coiling during replication. Irinotecan shows strong clinical antitumor effects by antagonizing DNA synthesis and is used widely for cancer treatment, particularly metastatic colorectal cancer, and for the treatment of solid tumors (3). Bone marrow suppression, such as neutropenia and thrombocytopenia, are well known as frequent severe adverse effects of irinotecan.

Recent advances in cancer treatment have significantly increased life expectancy, especially for young patients. Several studies, including ours, suggest possible mechanisms involved in gonadal-toxicity induced by cancer chemotherapeutic agents such as vascular toxicity, oxidative toxicity in somatic or germ cells micro-environment, direct anti-mitotic toxicity and apoptosis of germ cells or endocrine somatic cells in the testis, inflammation caused by possible breach of blood follicular or testicular barrier, chemotherapy-induced signal transduction leading to dedifferentiation of Sertoli cells or down-regulation of testosterone secretion by Leydig cells, and burn-out of follicular reserve by intra-ovarian cascade initiated by the activation and depletion of growing follicles (4–19). We have previously demonstrated that different classes of chemotherapeutic drugs display variable degrees and modes of gonadal toxicity. Due to the lack of evidence regarding gonadal effect of irinotecan, we aimed to evaluate irinotecan *in vivo* gonadotoxicity in a mouse model.

## Materials and Methods

### Experimental Design in Mice

Mature ICR male and female mice (2 months old; Envigo, Jerusalem, Israel), five in each group, were housed in air-conditioned, light-controlled animal facilities of the Sackler Faculty of Medicine in Tel-Aviv University. Our experience with ICR strain of mice in anti-cancer treatments and gonadal toxicity, provided us the knowledge and perspective to assess the relative severity of irinotecan treatment on the gonads (7, 10–12, 14, 15, 20). ICR mice are one of the most popular strains of outbred mice used in in pharmacology and oncology studies. This strain have been used extensively in toxicology and pharmacology studies and is often used for product safety testing (21–26). Animal care and all experiments were in accordance with institutional guidelines and were approved by the Institutional Animal Care and Use Committee, Sackler Faculty of Medicine, Tel-Aviv University, ID TAU-R 100106. Mice were weighted, injected intraperitoneally with either saline, irinotecan (100 mg/kg; CPT-11; Teva Pharmaceutical Industries, Petah Tikva, Israel) or cyclophosphamide (100 mg/kg; positive control for folliculogenesis; Endoxan; Baxter Oncology GmbH, Halle, Germany). Irinotecan non lethal, but highly effective treatment was according to Morishita et al. (2) and Lucić Vrdoljak et al. (27). Cyclophosphamide dose and administration method were according to Xie et al. (28) and Park et al. (29). Animals were euthanized with Isoflurane (Pharmal Healthcare, India) 1 week or 3 months later. Ovaries and testes were excised, weighed and further processed. Epididymides were also excised and weighed for assessment of spermatogenesis. Cauda epididymides were punctured and sperm were allowed to swim into M2 medium (M-7167; Sigma Chemical, St. Louis, MO, USA) at 37°C in 35 mm Petri dishes. Sperm concentration and motility were assessed by Makler counting chamber (Sefi Medical Instruments, Haifa, Israel).

### Enzyme-Linked Immunosorbent Assay for AMH

Blood was drawn from the inferior vena cava of mice. Samples were centrifuged (6,000 rpm, 10 min, 4°C) and sera were stored at −80°C. AMH levels were determined by ELISA AMH gen II kit (Beckman Coulter, Chaska, MN, USA) according to the manufacturer's instructions and served as marker for both ovarian reserve and testicular toxicity (10).

### Immunohistochemistry, Immunofluorescence, Terminal Transferase-Mediated Deoxyuridine 5-Triphosphate Nick-End Labeling and Histomorphometric Computerized Analysis of Testes and Ovaries

Sections of paraffin-embedded testes and ovaries were processed for IHC as previously described (11) with the following primary antibodies: rabbit anti-Ki-67 (1:300; Spring Bioscience, CA, USA; E1871), rabbit anti-proliferating cell nuclear antigen (PCNA; 1:100; Santa Cruz Biotechnology, Santa Cruz, CA, USA; sc-7907) and rat anti-cluster of differentiation (CD34; 1:100; Cedarlane, Ontario, Canada; CL8927AP). We used Hoechst 33280 (1 μg/ml; Sigma Chemical) for DNA staining, mixed with the following secondary antibodies: HRP-conjugated donkey anti-rabbit (1:200; Abcam, Cambridge, MA, USA; ab16284), Alexa-488-conjugated donkey anti-rabbit (1:200; Abcam; ab150073), Alexa-555-conjugated donkey anti-rat (1:200; Abcam; ab150154). DNA fragmentation was examined by TUNEL according to manufacturer's instructions (Dead End fluorometric TUNEL system; Promega, Madison, WI, USA). Saline group sections were exposed for 10 min to DNase I (6 units/ml; Invitrogen, Carlsbad, CA, USA). Bright-field images were recorded by a digital-camera (Canon pc1089 CCD, Tokyo, Japan) connected to an Axiovert 200M inverted microscope (Carl Zeiss MicroImaging; Oberkochen, Germany) equipped with an Apochromat 20X objective. Florescence images were photographed by LSM-510 confocal laser-scanning microscope (CLSM; Carl Zeiss MicroImaging) equipped with Plan-Neofluar 25X objective. Offset calibration of the photomultiplier was performed with sections stained with secondary antibodies only. Ki-67 staining of tonsil tissue served as positive control for immunoperoxidase staining. Twenty randomly selected transverse sections of testes or ovaries of three mice from each experimental group and from each immunostaining were used for analysis. The average number of Ki-67 positive cells, PCNA positive cells, TUNEL positive cells or CD34 positive staining area for quantifying incidence of blood vessels were automatically analyzed by Fiji software (National Institutes of Health, Bethesda, USA). The average numbers of primordial, primary, secondary and antral follicles were counted as previously described (20).

### Quantitative Real-Time PCR (qPCR)

Mice testicular and ovarian RNAs were isolated and quantified (14); first-strand cDNA was created by RT (Applied biosystems, Foster City, CA, USA) in 35 cycles with 0.4 μM gene-specific primers using ready-mix mixture (Sigma Chemical). The amount of mRNA was assessed by SYBR green reagent (SYBR Green PCR Master Mix, ABI, Carlsbad, CA, USA) on an ABI Prism 7900 Sequence PCR machine. In each run, 20 ng of cDNA per reaction were used as an amplification template and the primers used were as follows: mouse inhibitor of differentiation 4 (Id4) forward 5′ AGGGTGACAGCATTCTCTGC 3′; mouse Id4 reverse 5′ CCGGTGGCTTGTTTCTCTTA 3′; mouse GNDF family receptor alpha-1 (Gfra1) forward 5′ GCGTGTGAAGCACTGAAGTC 3′; mouse Gfra1 reverse 5′ GGTTCAGTTCCGACCCAAC 3′; mouse spermatogenesis- and oogenesis-specific basic helix-loop-helix transcription factor 2 (SOHLH2) forward 5′ TCTCAGCCACATCACAGAGG 3′; mouse SOHLH2 reverse 5′ GGGGACGCGAGTCTTATACA 3′; mouse newborn ovary homeobox gene (NOBOX) forward 5′ CATGAAGGGGACCTGAAGAA 3′; mouse NOBOX reverse 5′ GGAAATCTCATGGCGTTTGT 3′; mouse factor in the germline alpha (FIGLA) forward 5′ ACAGAGCAGGAAGCCCAGTA 3′; mouse FIGLA reverse 5′ TGGGTAGCATTTCCCAAGAG 3′. The house-keeping gene selected for the qPCR calibration was hypoxanthine-guanine phosphoribosyl transferase (HPRT1) and the primers used were as follows: HPRT1 forward 5′ CTCATGGACTGATTATGGACAGGAC 3′; mouse HPRT1 reverse 5′ GCAGGTCAGCAAAGAACTTATAGCC 3′. Data was recorded and analyzed by StepOne 2.1 software (Applied biosystems, ThermoFisher Scientific, USA).

### Statistical Analysis

Quantitative measurements are presented as mean ± standard error of mean (SEM). Data were evaluated by independent, two-sample *t*-test for unequal sample sizes and unequal variances with significance of *P* < 0.05. A correlated one-way ANOVA statistical analysis showed similar results.

## Results

### Irinotecan-Induced Ovarian Toxicity

**Figure 1:**
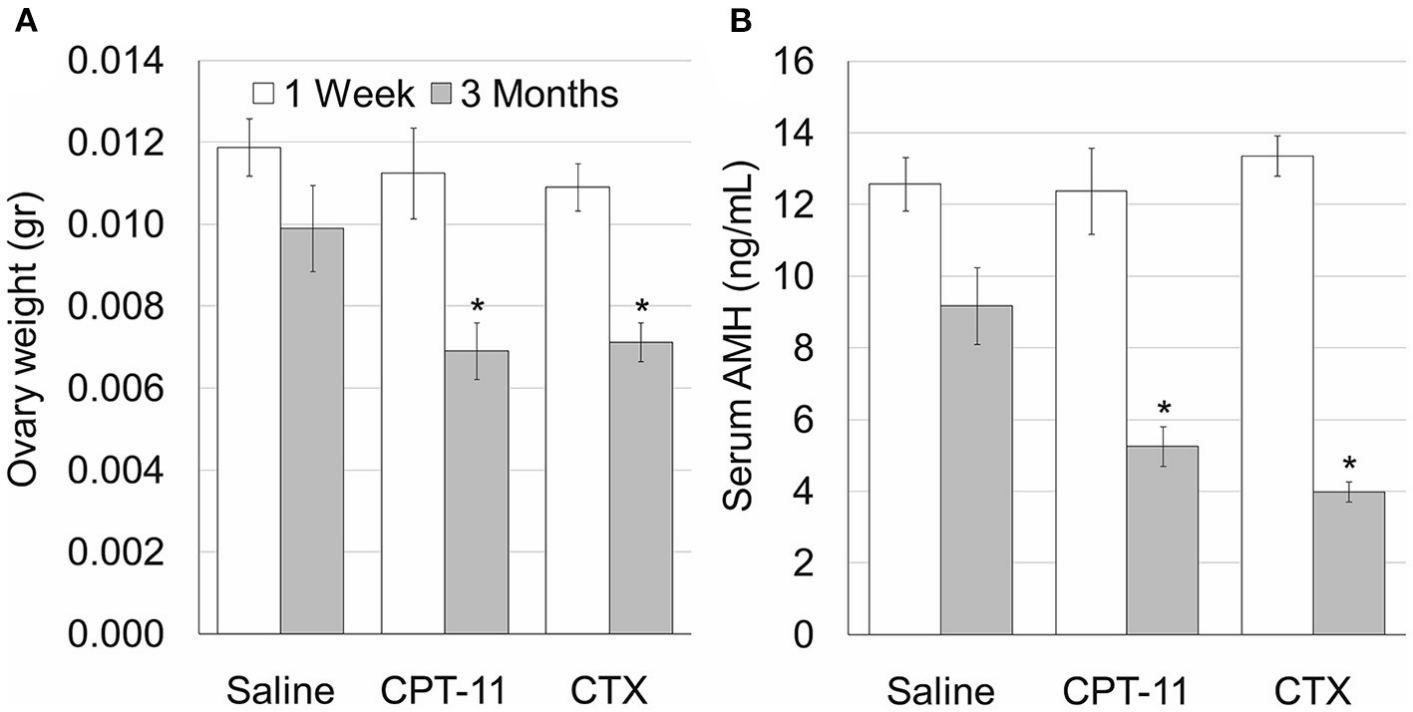
Irinotecan mild ovarian toxicity. Mature female mice (2 months old) were weighted, injected intraperitoneally with saline, irinotecan (100 mg/kg; CPT-11) or cyclophosphamide (100 mg/kg; CTX). Mice were sacrificed 1 week (5, 5 and 5 mice, respectively; white bars) or three months (4, 5 and 5 mice, respectively; gray bars) later. Ovary weight **(A)** and Serum AMH **(B)** were measured. Bars are mean ± standard error of mean. (*), significantly different from saline value (*P* < 0.05). CPT-11, irinotecan; CTX, cyclophosphamide.

**Figure 2:**
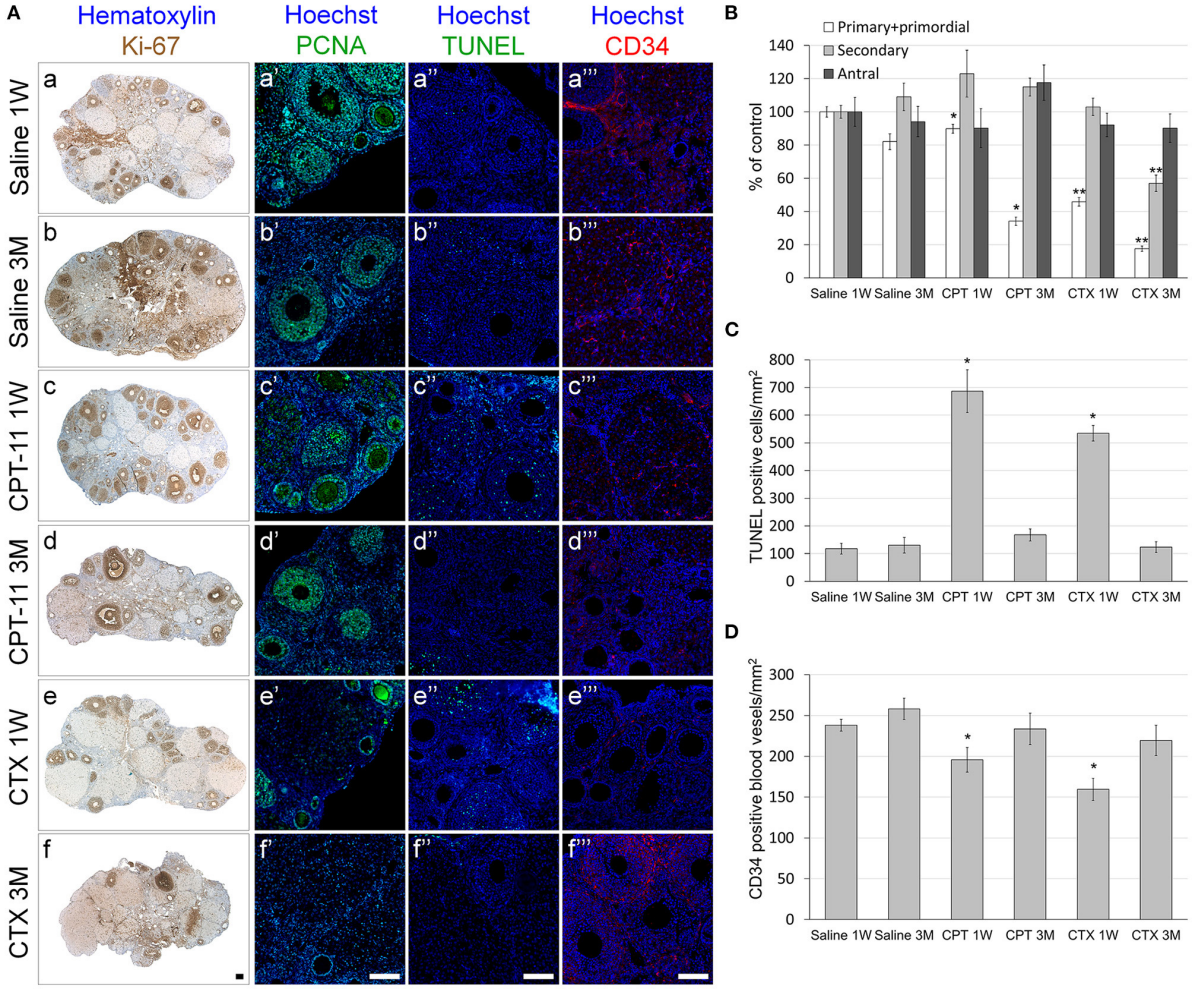
Ovarian follicles, proliferation, apoptosis and vascular state in mice after irinotecan treatment. Mature female mice were treated as described in the legend of Figure 1. Ovaries were excised from mice 1 week (1W) or 3 months (3M) after treatment, fixed, paraffin-embedded and serially sectioned for immunohistochemistry, immunofluorescence and terminal transferase-mediated deoxyuridine 5-triphosphate nick-end labeling (TUNEL) assay. **(A)** Representative bright field images of ovaries stained with Ki-67 (brown; **A**a-f) and representative florescence images of ovaries stained anti-PCNA (green; **A**a'-f'), TUNEL (green; **A**a”-f”) or CD34 (red; **A**a”'-f”'). Bars = 100 μm. Twenty randomly selected transverse sections of ovaries of three mice from each experimental group, each staining and each time point (1 week, white bars; 3 months, gray bars) were used for automatic analysis by Fiji software. The average amount of primordial and primary (white bars), secondary (gray bars) and antral (black bars) follicles **(B)** and the average amount of ovarian TUNEL positive cells/mm^2^ as a measure of apoptosis **(C)** and CD34 positive blood vessels/mm^2^ as a measure of blood vessels vascularity **(D)** per ovary 1 week (1W) or 3 months (3M) after treatment were measured. Bars are mean ± standard error of mean. (*), significantly different from saline value (*P* < 0.05). (**), CPT-11 significantly different from CTX value (*P* <0.05). CPT-11, irinotecan; CTX, cyclophosphamide.

We examined the effect of irinotecan on mouse gonads, 1 week or 3 months after drug administration, for assessment of gonadal short- and long-term effects, respectively. First, we examined the effect on several conventional markers of ovarian function and on ovarian reserve, which are known to be affected by chemotherapy insult. We used cyclophosphamide, which is considered a prototype for gonadotoxic chemotherapy, as a reference. Our findings demonstrate a long-term decrease of ovarian weight (Figure 1A) and serum AMH level (an indirect indicator of ovarian reserve; Figure 1B) after irinotecan treatment, similar to the decrease induced by cyclophosphamide treatment (Figures 1A,B). Next, we assessed the specific effect of irinotecan on the morphology, apoptosis and vascularity of ovarian follicles, by means of immunohistochemistry, immunofluorescence and TUNEL assay. For these assays we used randomly selected transverse sections of ovaries from mice in each experimental group of each staining and of each time points. We used histomorphometric and automatic computerized analysis of the acquired images. We did not observe changes in proliferation per follicles, but a significant change in follicle count. In the following groups: Saline 1W, 3M, CPT-11-1W, 3M, CTX-1W and CTX-3M, number of positive Ki-67 cells in antral follicles were 102.5 ± 34.2, 96.4 ± 27.3, 110.9 ± 29, 104.6 ± 8, 96 ± 21.14, 103 ± 26.9 (mean ± SEM), respectively; number of positive Ki-67 cells in secondary follicles were 85.3 ± 25.8, 92.4 ± 23.1, 89.7 ± 26.8, 97.5 ± 30.7, 89.5 ± 31, 79.2 ± 24.6 (mean ± SEM), respectively; number of positive Ki-67 cells in primordial and primary follicles were 23.1 ± 6.5, 25.6 ± 8, 21.9 ± 7.3, 26.4 ± 9.5, 22.5 ± 7.6, 28.5 ± 9.4 (mean ± SEM), respectively. Our findings indicate short- and long-term decrease in the number of primary and primordial follicles, 1 week and 3 months after irinotecan treatment, respectively (Figures 2Ac,d,B). A greater decrease in the number of primary and primordial follicles was also observed 1 week and 3 months after cyclophosphamide treatment (Figures 2Ae,f,B), which also caused a significant long-term decrease in the number of secondary follicles. Neither irinotecan nor cyclophosphamide affected the total number of antral follicles. Additional qualitative examination showed that ovaries exhibit less Ki-67 or PCNA positive proliferating follicles, 3 months after irinotecan or cyclophosphamide treatment (Figure 2Ad,d',f,f').

**Figure 3:**
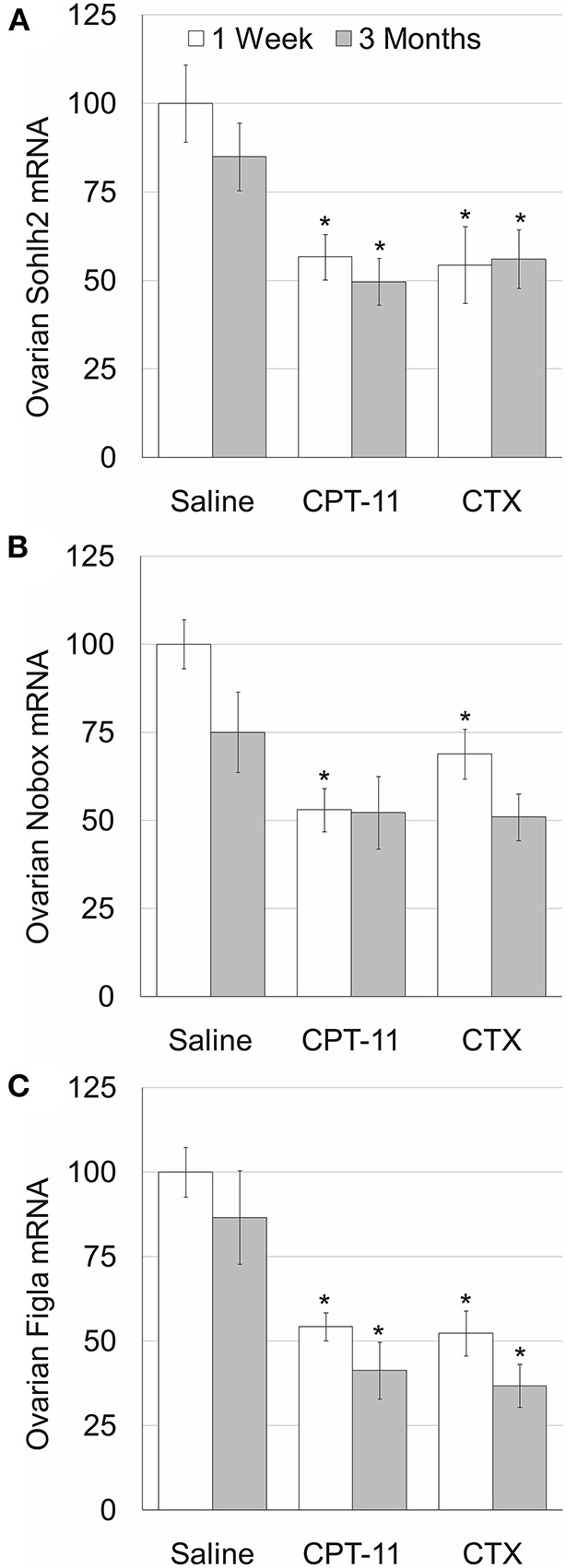
Ovarian reserve in mice after irinotecan treatment. Mature female mice were treated as described in the legend of Figure 1. Ovaries were excised from mice 1 week (white bars) or 3 months (gray bars) after treatment and ovarian SOHLH2 **(A)**, NOBOX **(B)** and FIGLA **(C)** mRNAs were measured. Bars are % of control ± standard error of mean. (*), significantly different from saline value (*P* <0.05). CPT-11, irinotecan; CTX, cyclophosphamide.

To examine whether the irinotecan-induced ovarian and follicular toxicity is caused by apoptosis or by vascularization damage, we employed TUNEL assay, as well as immunostaining and immunofluorescence of CD34, respectively. Our data demonstrate a similar degree of increased apoptosis (Figures 2Ac”,e”,C) and of reduced CD34-positive blood vessels (Figures 2Ac”',e”',D), 1 week after irinotecan or cyclophosphamide administration. Most apoptosis was found in granulosa follicular cells. Next, we examined the effect of irinotecan on the ovarian reserve, using quantitative real-time PCR of mRNA of key genes expressed primarily in oocytes of primordial follicles: Sohlh2, Nobox and Figla (direct indicator of ovarian reserve; 13). Our findings show a significant decrease of all three indicators 1 week and 3 months after irinotecan administration, with the exception of Nobox 3 months after irinotecan or cyclophosphamide administration (Figure 3). The observed irreversible effect of irinotecan on loss of ovarian reserve was similar to the effect observed after cyclophosphamide administration.

### Irinotecan-Induced Testicular Toxicity

**Figure 4:**
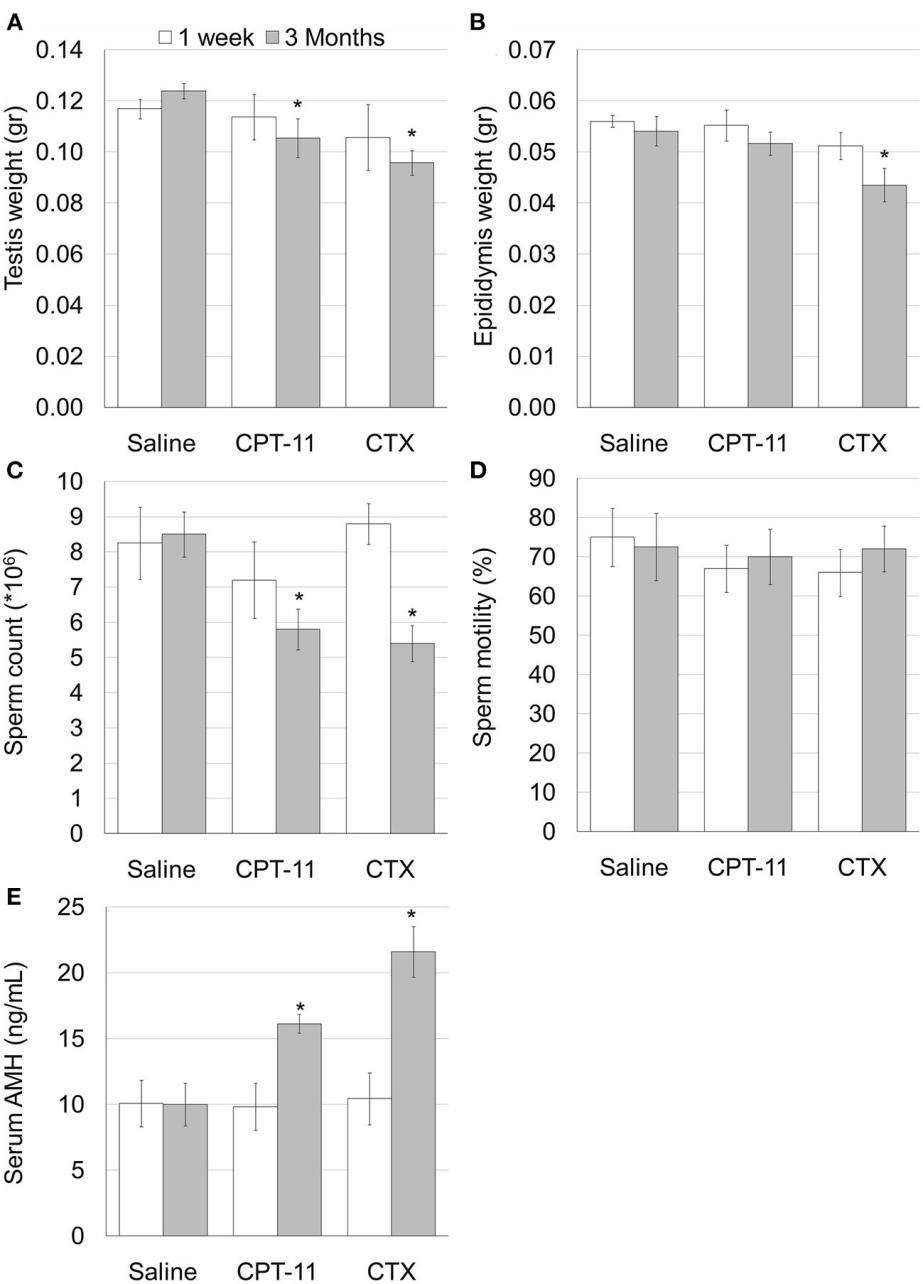
Irinotecan-mild testicular toxicity. Mature male mice (2 months old) were weighted, injected intraperitoneally with saline, irinotecan (100 mg/kg; CPT-11) or cyclophosphamide (100 mg/kg; CTX). Mice were sacrificed 1 week (4, 5, and 5 mice, respectively; white bars) or 3 months (4, 5 and 5 mice, respectively; gray bars) later. Testis weight **(A)** and epididymis weight **(B)**, epididymal sperm count **(C)**, sperm motility **(D)** and serum AMH **(E)** were measured. Bars are mean ± standard error of mean. (*), significantly different from saline value (*P* <0.05).

**Figure 5:**
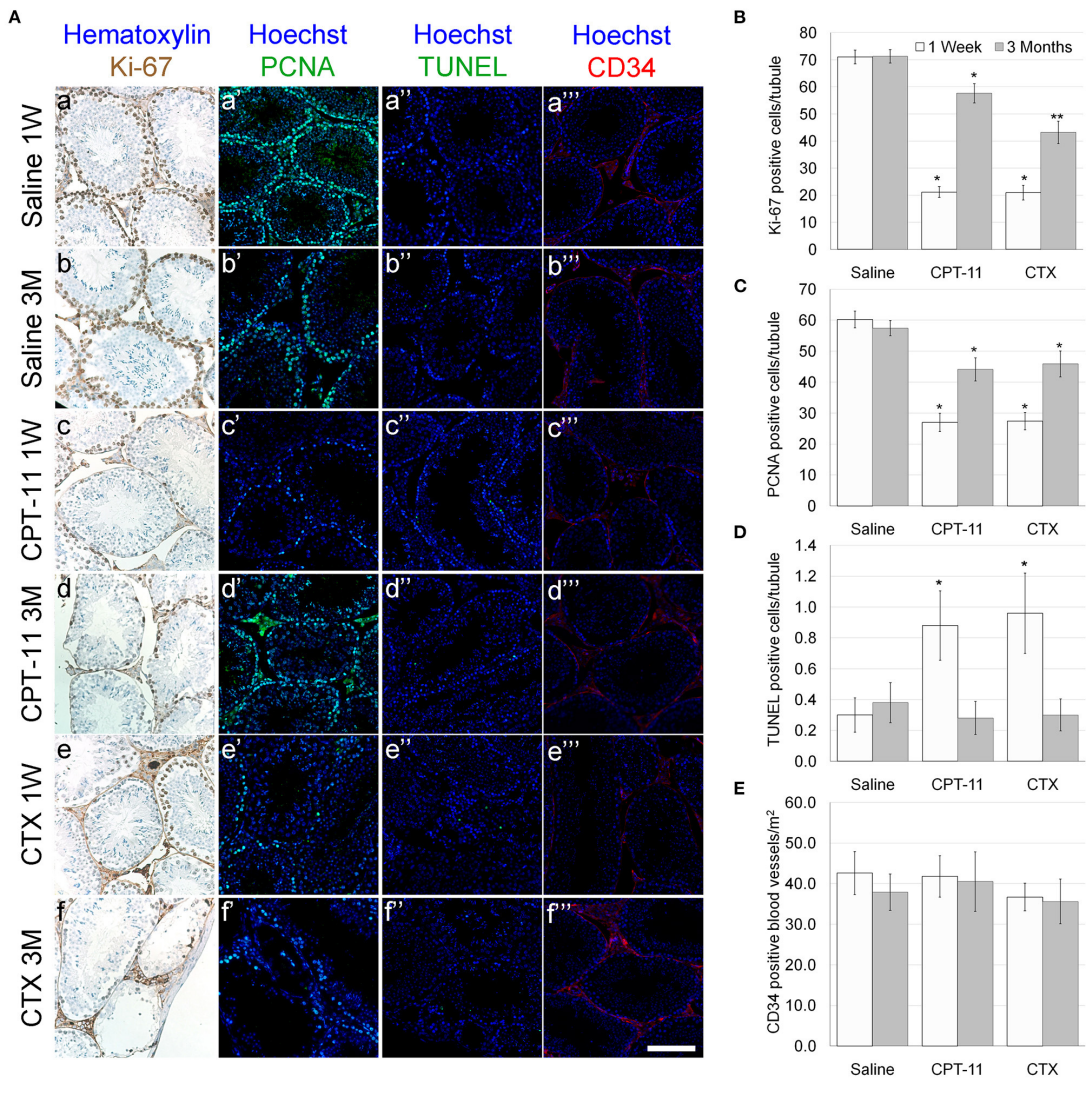
Testicular proliferation, apoptosis and vascular state in mice after irinotecan treatment. Mature male mice were treated as described in the legend of Figure 4. Testes were excised from mice 1 week (1W) or 3 months (3M) after treatment, fixed, paraffin-embedded and serially sectioned for immunohistochemistry, immunofluorescence and TUNEL assay. **(A)** Representative bright field images of ovaries stained with Ki-67 (brown; **A**a-f) and representative florescence images of ovaries stained anti-PCNA (green; **A**a'-f'), TUNEL (green; **A**a”-f”) or CD34 (red; **A**a”'-f”'). Bars = 100 μm. Twenty randomly selected transverse sections of testes of three mice from each experimental group, from each staining and each time point (1 week, white bars; 3 months, gray bars) were used for automatic analysis by Fiji software. The average number of Ki-67 **(B)** and PCNA **(C)** positive cells per seminiferous tubule were used as a measure of proliferation. The average number of TUNEL positive cells per seminiferous tubule **(D)** was used as a measure of apoptosis and the average number of CD34 positive vessels per mm^2^ **(E)** was used as a measure of blood vessels vascularity. Bars are mean ± standard error of mean. (*), significantly different from saline value (*P* <0.05). (**), CPT-11 significantly different from CTX value (*P* <0.05). CPT-11, irinotecan; CTX, cyclophosphamide.

**Figure 6:**
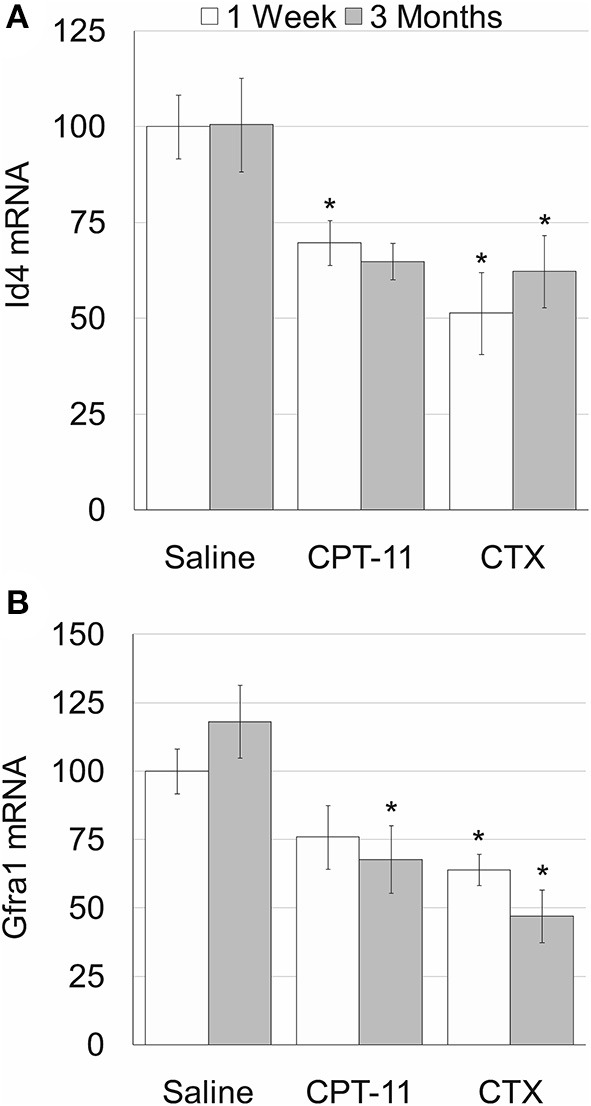
Testicular reserve in mice after irinotecan treatment. Mature male mice were treated as described in the legend of Figure 4. Testes were excised from mice 1 week (white bars) or 3 months (gray bars) after treatment and testicular Id4 **(A)** and Gfra1 **(B)** mRNA were measured. Bars are % of saline control ± standard error of mean. (*), significantly different from saline value (*P* <0.05). CPT-11, irinotecan; CTX, cyclophosphamide.

Our findings demonstrate that irinotecan treatment results in a moderate long-term effect on the testes, similar to the effect on the ovaries. Irinotecan administration decreased testicular weight (Figure 4A) and sperm count (Figure 4C) 3 months after drug administration. Interestingly, cyclophosphamide administration resulted in a similar effect on both parameters; but unlike irinotecan, it also reduced epididymal weight (Figure 4B). Both drugs had no effect on sperm motility (Figure 4D), but caused a moderate increase in serum AMH level (Figure 4E). We assessed the specific effect of irinotecan on testicular function by examining spermatogenesis, apoptosis and vascularization using immunohistochemistry, immunofluorescence and TUNEL assay as well as automatic computerized analysis of randomly selected testicular sections. Our findings indicated a transient decrease in markers of proliferation (Ki67 and PCNA) in the testis 1 week and 3 months after irinotecan treatment (Figures 5Ac,d,c',d',**B,C**). Irinotecan treatment caused a transient increase in apoptosis (Figures 5Ac”,**D**), but had no effect in the amount of CD-34 positive blood vessels (Figures 5Ac”',d”',**E**). Apoptosis was found in dividing germ cells such as spermatogonia and spermatocytes. Cyclophosphamide treatment displayed similar effect on the abovementioned parameters, but showed a more significant decrease in Ki-67 level. Positive proliferating cells were almost absent from seminiferous tubules 3 months after drug administration (Figures 5Af,**B**). Finally, we used qPCR to measure mRNA of transcription factors indicating undifferentiated spermatogonia, namely Id4 and Gfra1, which serve as markers of testicular reserve (14). The amount of Id4 decreased significantly 1 week after irinotecan administration (Figure 6A), whereas the amount of Gfra1 decreased 3 months after irinotecan administration (Figure 6B). The irreversible effect on testicular reserve loss was similar to the effect observed after cyclophosphamide administration.

## Discussion

Due to the rising incidence of specific cancer types in young population, the issue of gonadal effect of anti-cancer treatments becomes highly relevant for better patient counseling regarding fertility preservation. Future development is necessary to minimize gonadal toxicity. The high growth rate of the germ cell population in testis and somatic cells in ovary renders the gonads particularly sensitive to chemotherapeutic drugs whose principal mechanism of action is to impair the replication ability of cancer cells (1). Irinotecan, a commonly used chemotherapy is an inhibitor of topoisomerase I, and as such, impairs cell proliferation. The long-term effect of this drug on gonadotoxicity and fertility has not been described. *In vitro* assay in testicular seminiferous tubules of prepubertal mice showed that the irinotecan metabolite SN38, at concentrations that are relevant to cancer patients, reduces germ cell number and proliferation, whereas it has little if any effect on the female germ cell population (30). When mice prepubertal ovaries were treated with SN38, no effect was observed in germ cells number, apoptosis or cell proliferation (30). Clinical results indicate that repeated irinotecan administrations may frequently induce acute ovarian follicular loss and premature ovarian failure, even in young women (1). Other studies showed that irinotecan causes acute granulosa cell-specific apoptosis partly through Fas ligand interactions in mice ovaries (1, 31, 32). In accordance with the publications mentioned above, our findings in the preclinical setting demonstrates, a mild and partial, but irreversible, long-term decrease of proliferating follicles, ovarian weight, serum AMH and ovarian reserve after irinotecan treatment in mice. This effect on mouse ovary may be the result of acute ovarian apoptosis and reduction of blood vessels, observed 1 week after irinotecan treatment (33).

During normal follicular development, the ovary is in a state of equilibrium. Exposure to ovotoxic agents, disturb this balance by destroying or removing growing follicles and activating the PI3K/PTEN/Akt follicle activation pathway. This reduces the negative suppression of the dormant PMF population, triggering increased recruitment of primordial follicle, causing the reservoir to burnout (8, 9, 34, 35). Our findings suggest that burn out is also the mechanism of action of irinotecan that reduces the number of primordial follicles mainly 3 months after drug administration (long term effect). Our study indicates that irinotecan treatment resulted in a moderate long-term effect in mice testes. Irinotecan administration reduced testicular weight, sperm count and testicular reserve and increased serum AMH, which is considered a surrogate reciprocal biomarker for testicular toxicity (10). Interestingly, irinotecan treatment transiently elevated apoptosis in testes and reduced spermatogenesis, but did not affect the amount of blood vessels, implying that its testicular toxicity mechanism of action does not include vascular injury. The reference cyclophosphamide treatment resulted in a similar moderate effect in both genders. Stem spermatogonia are damaged by chemotherapeutic agents at varying degrees and recover only gradually, resulting in prolonged reductions in sperm count. Stem spermatogonia cells represent the testicular reserve and play important part in testicular recovery and repopulation after induced damage. Several studies showed that the stem spermatogonia are more sensitive than the differentiating germ cells to the long-term effects of chemotherapy (36). Anti-cancer agents as irinotecan and cyclophosphamide, highly affect actively dividing cells. It has been shown that apoptosis of germ cells is a major mechanism of testicular damage induced by chemotherapy (10, 37). Irinotecan derivate, SN38, targeted spermatogonia *in vitro* in prepubertal mouse testis, and markedly reduced germ cells number and increased Sertoli cell-only tubules. The mechanism of irinotecan-induced gonadotoxicity could include factors intrinsic to the germ cells or indirect actions via somatic cells that impair signaling to germ cells, resulting in germ cells loss. Observations of indirect effects of other chemotherapeutic agents on spermatogonia proliferation and differentiation via their effect on testicular specific somatic cells, such as Sertoli cells, Leydig cells or peritubular myoid cells (30), suggest that irinotecan may also cause indirect damage to testicular reserve through its toxicity to these somatic cells. Moreover, our observation that irinotecan causes vascular damage in the ovaries, but not in the testes, also strengthens the hypothesis of distinct mechanisms of irinotecan-induced gonadotoxicity that are gonad-driven. Vascular toxicity, a major factor of chemotherapy-induced ovarian toxicity (7, 38) may play an important part in ovarian irinotecan toxicity. The evolution of vasculature in the gonads is differential between ovaries and testes. Whereas, ovaries recruit vasculature by a typical angiogenic process, testes recruit and patterns vasculature by a novel remodeling mechanism, beginning with breakdown of an existing mesonephric vessel followed by individual endothelial cells that migrate and re-aggregate in the coelomic domain to form the major testicular artery (39). Furthermore, physiological function of adult ovary and uterus depends on neovascularization (39). The use of a milder gonadotoxic regimen for adjuvant or neoadjuvant chemotherapy may be considered in young cancer patients with favorable tumor stages who are in their reproductive years (33). In conclusion, our study indicates mild ovarian toxicity and moderate testicular toxicity induced by irinotecan. Further studies are warranted in the clinical setting to validate current findings for tailored patient counseling for infertility risks and fertility preservation in cancer patients.

## Data Availability Statement

The raw data supporting the conclusions of this article will be made available by the authors, without undue reservation.

## Ethics Statement

The animal study was reviewed and approved by Institutional Animal Care and Use Committee, Sackler Faculty of Medicine, Tel-Aviv University, ID TAU-R 100106.

## Author Contributions

IB-A and ML designed the experiments and wrote the manuscript. ML had carried out the experiments. RS participated in the design and coordination, helped drafting the manuscript, and supervised the study. All authors read and approved the final manuscript.

## Funding

This work was supported by a grant from the Israel Science Foundation [Grant Number 1816/13 to IB-A].

## Conflict of Interest

The authors declare that the research was conducted in the absence of any commercial or financial relationships that could be construed as a potential conflict of interest. The reviewer AB declared a shared affiliation, with several of the authors ML and RS to the handling editor at the time of the review.

## Publisher's Note

All claims expressed in this article are solely those of the authors and do not necessarily represent those of their affiliated organizations, or those of the publisher, the editors and the reviewers. Any product that may be evaluated in this article, or claim that may be made by its manufacturer, is not guaranteed or endorsed by the publisher.
